# Screening of Various Metabolites in Six Barley Varieties Grown under Natural Climatic Conditions (2016–2018)

**DOI:** 10.3390/microorganisms7110532

**Published:** 2019-11-06

**Authors:** Kristina Habschied, Rudolf Krska, Michael Sulyok, Bojan Šarkanj, Vinko Krstanović, Alojzije Lalić, Gordana Šimić, Krešimir Mastanjević

**Affiliations:** 1 Faculty of Food Technology Osijek, Josip Juraj Strossmayer University of Osijek, F. Kuhača 20, 31000 Osijek, Croatia; kristinahabschied@gmail.com (K.H.); vkrstano@ptfos.hr (V.K.); kmastanj@gmail.com (K.M.); 2 Institute of Bioanalytics and Agro-Metabolomics, Department for Agrobiotechnology (IFA-Tulln), University of Natural Resources and Life Sciences, Vienna (BOKU), Konrad–Lorenz-Straße 20, 3430 Tulln an der Donau, Austria; rudolf.krska@boku.ac.at (R.K.); michael.sulyok@boku.ac.at (M.S.); 3 Institute for Global Food Security, School of Biological Sciences, Queens University Belfast, University Road, Belfast BT7 1NN, Northern Ireland, UK; 4 Department of Food Technology, University North, University Center Koprivnica, Trg dr. Žarka Dolinara 1, 48000 Koprivnica, Croatia; 5 Agricultural Institute Osijek, Južno predgrađe 17, 31000 Osijek, Croatia; alojzije.lalic@poljinos.hr

**Keywords:** metabolites, climate changes, barley crop

## Abstract

Climatic changes influence considerably the distribution and occurrence of different secondary metabolites in cereals. The aim of this investigation was to assess the changes in metabolite prevalence observed in six different winter barley varieties over a statistically significant period of three years by linking agro-climatic conditions with metabolite concentrations in chosen samples. The results showed that temperatures and precipitation levels varied during the observed timeframe and that the multi-toxin concentrations followed the trend of changing climatic conditions depending on the variety. All quantified (fungal) metabolites showed significant variations throughout the years and, for some (tryptophol and the cyclic dipeptides cyclo(L-Pro-L-Tyr) and cyclo(L-Pro-L-Val)), an unexpected, but clear connection can be made with temperature changes and precipitation levels during the growing season.

## 1. Introduction

Barley (*Hordeum vulgare* L.) is the main malting and brewing cereal, grown globally. Changes in climate greatly influence the microbial diversity of cereals. Shifts of *Fusarium* species have already been reported by several authors across the globe [1,2,3]. It is known that certain *Fusarium* species are affected by the rising temperatures and can be considered as an indicator of global warming. *Fusarium culmorum* is one of the eclectic examples of this phenomenon. This fungus was rather usual in Central and Eastern European countries, but with global warming this fungus is hard to be found in these parts of Europe, and even Northern Europe experts report its reduction. The more prevalent species, *Fusarium graminearum*, has taken its place and is spreading across the increasingly warmer European content [1]. The shift in *Fusarium* species certainly indicates the shift in all microbial life forms, and this purports the hypothesis that the secondary metabolites that these microorganisms produce are probably undergoing some changes as well. Janhager [4] conducted a research study on Swedish cereals and claims that weather conditions have the most influence on the increase in multi-toxin concentrations. Mycotoxins and plant toxins, or rather metabolites, are regularly detected in cereals. Some are regarded as toxic for humans and animals and clear legislative limits have been set by the European Union (EU) [5] in order to keep them under control. Some, however, are still not recognized by the legislative institutions and represent danger to human health. These are mostly conjugated toxins that are transformed during digestion, food processing, or via plant enzymes into the modified form (glucosides, sulphates, acetyl forms, etc.) or the conjugated form gets degraded into the original molecule. In some cases, both forms regularly occur together, such as deoxynivalenol (DON) and its acetylated forms 3- and 15-acetyldeoxynivalenol (3- and 15-AcDON) [6]. Emerging toxins are yet to be investigated regarding their toxicity to human and animal health, and then included in legislative procedures. In any case, novel or emerging mycotoxins or plant toxins, modified or basic forms, are an important factor in the sustainable food chain.

The aim of this research was to assess the content of different metabolites in cereals over a statistically significant period of three years, in six different barley varieties in regards to micro-climatic conditions during the growing season. Our hypothesis was that the metabolite concentrations would be higher in years with extreme temperatures or precipitation.

## 2. Materials and Methods

### 2.1. Barley Samples

Six winter hulled two-rowed barley varieties (*Hordeum vulgare* L. *sensu lato*, *conv. distichon*) with different end-use purpose: Barun (dual), Bingo (feed), Bravo (feed), Lukas (dual), Maxim (dual), and Vanessa (brewing) were collected from trial fields of the Agricultural Institute in Osijek, Croatia, at location Osijek (45°32′N, 18°44′E). The soil pH was 6.25, and the type was eutric cambisol. Varieties tested in this research differ in the heading date: Bingo and Barun are early heading varieties, Lukas, Maxim, and Bravo are middle heading varieties, and Vanessa is a later heading variety. Barun, Bingo, Bravo, Lukas and Maxim are domestic varieties created at Agricultural Institute Osijek, Croatia. Variety Vanessa is traditional malting type barley, created at Saatzucht Josef Breun GmbH – Herzogenaurach breeding station in Germany. Dual purpose varieties refer to malting–feed varieties (they can be used as malting and/or feed varieties). Samples of six different varieties were collected over three consecutive seasons (2016–2018). Field experiments were conducted in randomized complete block designs (RCBD) with six replications; plot size was 7.56 m^2^. Soil properties and climatic conditions during the growing seasons (October–June) at the respective locations are displayed in Table 1. Sampling (200 g per sample) was performed on cleaned and processed barley grains and the samples were kept refrigerated in sterile dry containers.

May and June were isolated as the most important months for mycotoxin production. Weather conditions during flowering (May) and harvest (June) are the most significant factors influencing mycotoxins and other metabolites in barley. For that reason, Table 2 represents the statistical analysis of temperature and precipitation values in May and June for each year.

### 2.2. Analysis of Metabolites

Metabolites were determined according to Malachová et al. [7]. Analysis was performed in the Center for Analytical Chemistry, Department for Agrobiotechnology (IFA-Tulln) at the University of Natural Resources and Life Sciences, Vienna, Austria. In brief, a 5 g portion of the homogenized ground sample was extracted with extraction solvent consisting of acetonitrile:water:acetic acid = 79:20:1 for 90 min by using a GFL 3017 rotary shaker (GFL, Burgwedel, Germany) at 180 rpm and room temperature. Following extraction, a crude sample was precipitated and an amount of 500 μL of clear extract was diluted with dilution solvent (acetonitrile:water:acetic acid = 20:7:1). For the separation, an Agilent 1290 UHPLC system (Agilent, Santa Clara, CA, USA) was used combined with a Gemini^®^ C18 (150 × 4.6 mm i.d., 5 μm particle size) column, and C18 security guard cartridge, 4 × 3 mm i.d., whereas the Sciex 5500 QTrap^®^ system (Sciex, Framingham, MA, USA) was used for detection and quantification. All system parameters were as described in Malachová et al. [7] and all analysis were done in triplicate. Limit of detection (LOD) and limit of quantification (LOQ) values are shown in Table S1, Supplementary Materials.

### 2.3. Statistical Data Analysis

Experimental data were analyzed by the analysis of variance (ANOVA) and Fisher’s least significant difference (LSD), with significance defined at *p* < 0.05. Statistical analysis was carried out with Statistica 12.7 (2015, StatSoft Inc. Tulsa, OK, USA).

## 3. Results and Discussion

The results of detected metabolites are presented for each year and variety in Table 3 and Table 4. Although all toxins showed no excess values with respect to the EU legislation [5], the accumulation of different metabolic toxins appeared to be bound to climatic conditions, as previously reported by Janhager [4], and certain patterns can be established. Since deoxynivalenol is the most prevalent and most significant mycotoxin in cereals, this discussion starts with it. In general, during the growing season 2015–2016 when the temperature was lowest and precipitation was highest (Table 1), higher DON concentrations were expected. However, in this study, some varieties (Barun, Bravo, Maxim, and Vanessa) reacted differently and showed higher concentrations of DON in years with significantly higher temperatures during May and June (2017 and 2018) and lower precipitation (2017). Barun showed highest concentrations for DON in 2017, a warmer and dry year. This is in accordance with the research that Langseth and Elen conducted in 1997 [8] in Norway. Of all six varieties, Bingo accumulated the lowest concentrations of DON, with maximum value being 34 µg kg^−1^ in 2017. Bravo showed higher results regarding DON concentrations. Specifically, in 2018 Bravo had the highest concentration of DON, 86 µg kg^−1^, whereas in 2017 its concentration was significantly lower, 39 µg kg^−1^. Such low concentrations of DON (around 35 µg kg^−1^) in years with less rain during anthesis and the harvest period are in accordance with a study conducted by Langseth and Elen [8]. The two extremes regarding rain during May (27.3 mm) and June (126.6 mm) in 2018 (Table 2) clearly affected this variety. Although statistically different, the differences between years for DON in Lukas barley are not so far apart. During a three-year survey, Lukas showed a mean value for DON of about 72 µg kg^−1^, actually giving the best response to changes in precipitation and temperatures throughout the years. Maxim showed a significant discrepancy in DON concentration in 2017, a year marked by low precipitation and warm temperatures (107 µg kg^−1^), whereas Vanessa (a strict brewing variety) in 2017 showed the lowest value for DON (40 µg kg^−1^).

Alternariolmethylether-glucoside levels were mostly more pronounced in 2017 in Barun, Bravo, Maxim, and Vanessa, while DON-3-glucoside showed a similar trend in Bravo, Lukas, Maxim, and Vanessa for the same year. Other significant DON derivates, such as 15-acetyldeoxynivalenol and 3-acetyldeoxynivalenol, showed below LOD (limit of detection) or very low concentrations during the whole research period. Barun showed a maximal value of 25 µg kg^−1^ for 15-acetyldeoxynivalenol in 2017. Literature data refer to DON-3-glucoside concentrations as closely related to DON levels [9,10,11], but this appeared not to be the case in this research. Specifically, Tucker et al. [10] reported a very strong relationship between the ratio of DON-3-glucoside to DON. DON can be considered as a reliable predictor for total content of DON derivates (DON-3-glucoside, 15-acetyldeoxynivalenol, and 3-acetyldeoxynivalenol). In research conducted on them, the genotypes with the highest level of resistance showed an elevated ratio of DON-3-glucoside to DON. In matured grains, the ratio of DON-3-glucoside to DON was generally consistent for varieties, hence DON was considered a good predictor of all DON conjugates. Lemmens et al. [9] reported that FHB (Fusarium Head Blight) resistance reduces DON and DON-3-glucoside concentrations in the grain, but DON content is more subjected to reduction than DON-3-glucoside. This is best described by the fact that the DON-3-glucoside/DON ratio is on the increase, whereas DON is decreasing. In our research, the DON-3-glucoside/DON ratio showed no such correlation, rather a random set of data.

Culmorin was quantified in all samples. The highest concentrations were detected in 2017 in Lukas, Maxim, and Vanessa. In 2017, the lowest precipitation and modest temperatures were recorded. Barun, Bingo, and Bravo showed randomness regarding this compound. Barun had the highest concentration of culmorin in 2016, and Bingo and Bravo showed an increase in 2018. 

Lotaustralin is a cyanogenic glucoside whose biological role is to form a defense mechanism against various phytopathogens, even though animals, plants, and some fungi, including *Fusarium* species, are known to possess detoxifying abilities against these compounds [12,13]. Lotaustralin levels were heightened in some years, but this clearly depends on variety, since no variety reacted in the same way to climatic conditions.

Tryptophol is an aromatic higher alcohol [14] and growth hormone in plants. In this study, the highest concentrations of tryptophol were recorded in 2018 in all samples. This could indicate that this was a favorable year for growth and barley produced more tryptophol. In addition, this is a plant-based tryptophol (produced by plants) since all varieties showed an increase in the same year.

Unspecific cyclic dipeptides like cyclo(L-Pro-L-Tyr) and cyclo(L-Pro-L-Val) are yet to be pinpointed whether they originate from fungi or bacteria or from plants [15]. The concentrations of these dipeptides appeared to be the highest in 2016, in all samples. This is peculiar, but for that reason, and as for tryptophol, it can be assumed that, in this case, they are plant metabolites rather than fungal or bacterial.

Other significant mycotoxins commonly found in cereals, T-2 and HT-2, showed below LOD or very low concentrations during the whole research period. HT-2 toxin was quantified in very low values in Lukas (2017; 2.61 µg kg^−1^) and Vanessa (2018; 4.57 µg kg^−1^). T-2 was also detected in low concentrations for all varieties for all years, reaching the maximal concentration (13.1 µg kg^−1^) in 2018, a warmer year with precipitation extremes, in Vanessa. Previous reports correlated higher levels of precipitation with HT-2 and T-2 contamination [16].

Doohan et al. [17] stated that the conditions favorable for fungal growth are commonly also favorable for mycotoxin production on cereal grains. Results obtained in a research study by Schöneberg et al. [18] indicate that the optimum temperature for *F. graminearum* infection of barley is 15 °C.

There is also a certain correlation between CO_2_ levels in the atmosphere and mycotoxin production [19]. Determining the influence of climate on mycotoxin and other toxin production is a complex task, since the production of different toxins is influenced differently by the same weather conditions, as can be seen from this research. Even though the climate–toxin production shows a certain correlation, it seems very unpredictable and random. Since barley is the main commodity for malting and brewing, it is important to know the spectrum of metabolites that can be found in this cereal.

## 4. Conclusions

In order to pinpoint the actual changes brought about by climatic fluctuations, a more complex and longer-lasting monitoring should be conducted. The timespan in which the data are represented is short, but the values can point to a certain trend and can be used as a starting point for future and regular monitoring. Although this research showed that metabolites concentrations are not worryingly high and are inside the legislative limits for unprocessed cereals, the trend of an increase (or decrease) can be well established and it can be expected that the exposure to multi-toxins will rise due to climatic changes. The adverse effects of exposure to a combination of toxins are not completely known and this represents an additional threat to food production and, consequently, human and animal health. Climate changes affect the entire globe, putting food production at serious risk if no actions are undertaken. Multi-toxins should be taken seriously as they are the emerging poison of this civilization.

## Figures and Tables

**Table 1:** Precipitation and mean temperature during the growing season of winter barley (October–June).

	Mean Precipitation (mm)	Total Precipitation (mm)	Mean Temperature (°C)
2015–2016	66.11	594.99	8.3
2016–2017	46.20	415.83	9.1
2017–2018	60.43	543.90	10.2

**Table 2:** Statistical analysis of the temperature and precipitation in May and June for each year.

	2016	2017	2018
**Temperature (°C)**			
**May**	16.35^c^	17.35^b^	20.05^a^
**June**	21.15^b^	22.25^a^	20.09^b^
**Precipitation (mm)**			
**May**	63.0^a^	50.5^b^	27.3^c^
**June**	99.3^b^	45.3^c^	126.6^a^

Values in the same row with different letters a–c are significantly different (*p* < 0.05).

**Table 3:** Concentrations of other positively identified mycotoxins and fungal metabolites in barley samples.

Toxin (µg kg^−1^)	Barun (Dual)			Bingo (Feed)			Bravo (Feed)		
	2016	2017	2018	2016	2017	2018	2016	2017	2018
15-Acetyldeoxynivalenol	<LOD	25.37^a^	<LOD	<LOD	<LOD	<LOD	<LOD	<LOD	<LOD
15-Hydroxyculmorin	2.57^b^	1.40c	9.19^a^	1.64^a^	1.38^c^	1.59^b^	10.5^a^	3.31^b^	1.24^c^
15-Hydroxyculmoron	27.4^a^	<LOD	<LOD	1.51c	57.7^a^	12.4b	<LOD	<LOD	<LOD
3-Acetyldeoxynivalenol	<LOD	<LOD	<LOD	<LOD	<LOD	<LOD	<LOD	<LOD	<LOD
5-Hydroxyculmorin	42.1^a^	<LOD	<LOD	<LOD	<LOD	<LOD	<LOD	<LOD	<LOD
Alternariol	0.59^b^	0.86^a^	<LOD	<LOD	<LOD	<LOD	<LOD	<LOD	0.71^a^
Alternariolmethylether	0.47^a^	<LOD	<LOD	<LOD	<LOD	<LOD	<LOD	<LOD	<LOD
Alternariolmethylether-glucoside	24.8^b^	24.3^c^	28.8^a^	14.2^b^	21.9^a^	<LOD	10.8^b^	7.90c	25.3^a^
Brevianamid	10.6^a^	4.35^c^	8.41^b^	9.86^a^	5.41^b^	<LOD	12.5^a^	9.28^b^	5.46^c^
Citreorosein	9.21^a^	8.38^b^	0.83^c^	5.52^a^	0.83^b^	<LOD	2.28^a^	<LOD	1.99^b^
Culmorin	6.72^b^	1.20^c^	17.60^a^	11.8^a^	7.52^b^	2.94^c^	10.4^a^	4.67^b^	3.08^c^
Cyclo(L-Pro-L-Tyr)	21.1^a^	5.21^c^	12.5^b^	15.1^a^	9.29^b^	<LOD	25.4^a^	11.5^b^	5.61^c^
Cyclo(L-Pro-L-Val)	18.8^a^	3.40^c^	15.1^b^	20.1^a^	10.4^b^	<LOD	21.6^a^	14.9^b^	4.40^c^
Deoxynivalenol	55.5^c^	80.0^a^	57.9^b^	34.6^a^	24.9^b^	0.75^c^	63.5^b^	39.4^c^	86.0^a^
DON-3-glucoside	<LOD	<LOD	2.90^a^	<LOD	<LOD	<LOD	<LOD	1.73^a^	<LOD
Emodin	7.73^a^	4.37^b^	0.46^c^	4.87a	0.27^b^	<LOD	1.32^a^	<LOD	0.97^b^
Enniatin A	0.12^a^	<LOD	<LOD	<LOD	<LOD	<LOD	0.28^a^	<LOD	<LOD
Enniatin A1	1.46^a^	0.49^b^	<LOD	3.70^a^	<LOD	<LOD	1.35^a^	0.15^b^	<LOD
Enniatin B	9.54^a^	2.50^b^	<LOD	1.15^a^	<LOD	<LOD	0.43^b^	0.79^a^	0.11^c^
Enniatin B1	7.91^a^	3.72^b^	<LOD	4.70^a^	<LOD	<LOD	1.57^a^	0.86^b^	<LOD
Enniatin B2	0.33^a^	0.16^b^	<LOD	<LOD	<LOD	<LOD	<LOD	<LOD	<LOD
Fumonisin B1	<LOD	7.86^a^	7.10^b^	7.32^a^	6.01^b^	<LOD	10.9^a^	9.91^b^	6.74^c^
HT-2 toxin	<LOD	<LOD	<LOD	12.3^a^	<LOD	<LOD	<LOD	<LOD	<LOD
Lotaustralin	8.90^b^	9.08^a^	7.71^c^	8.46^a^	7.24^b^	6.41^c^	8.40^b^	8.13^c^	9.31^a^
Moniliformin	<LOD	<LOD	<LOD	0.38^b^	2.16^a^	<LOD	2.13^b^	2.48^a^	1.88^c^
Monocerin	2.79^a^	<LOD	<LOD	0.63^a^	<LOD	<LOD	0.31^a^	0.12^b^	<LOD
Rugulusovin	2.30^a^	0.99^b^	0.94^c^	1.05^a^	0.54^b^	<LOD	1.80^a^	0.64^c^	1.19^b^
T-2 toxin	<LOD	<LOD	<LOD	<LOD	<LOD	<LOD	<LOD	0.80b	1.48
Tentoxin	0.17^a^	0.11^b^	0.08^c^	0.30^a^	0.24^b^	<LOD	0.47^a^	0.28^b^	<LOD
Tryptophol	69.1^a^	31.3^c^	33.4^b^	37.6^b^	38.0^a^	2.51^c^	84.0^a^	45.4^b^	29.3^c^

Values are means of triplicate. Values in the same row (2016–2018 for each variety) with different letters a–c are significantly different (*p* < 0.05). LOD and LOQ values are shown in Table S1, Supplementary Materials.

**Table 4:** Concentrations of other positively identified mycotoxins and fungal metabolites in barley samples.

Toxin (µg kg^−1^)	Lukas (Dual)			Maxim (Dual)			Vanessa (Brewing)		
	2016	2017	2018	2016	2017	2018	2016	2017	2018
15-Acetyldeoxynivalenol	<LOD	<LOD	<LOD	<LOD	<LOD	<LOD	<LOD	<LOD	<LOD
15-Hydroxyculmorin	5.69^b^	17.6^a^	1.70^c^	5.27^a^	5.14^b^	3.99^c^	4.19^b^	8.60^a^	1.85^c^
15-Hydroxyculmoron	<LOD	<LOD	<LOD	<LOD	<LOD	<LOD	<LOD	33.44^a^	<LOD
3-Acetyldeoxynivalenol	<LOD	<LOD	<LOD	<LOD	<LOD	<LOD	<LOD	<LOD	<LOD
5-Hydroxyculmorin	<LOD	<LOD	<LOD	59.9^a^	<LOD	<LOD	<LOD	<LOD	<LOD
Alternariol	1.50^b^	<LOD	8.61^a^	<LOD	<LOD	8.52^a^	<LOD	<LOD	4.74^a^
Alternariolmethylether	<LOD	<LOD	3.90^a^	<LOD	<LOD	2.51^a^	<LOD	<LOD	0.75^a^
Alternariolmethylether-glucoside	11.4^c^	12.5^b^	24.1^a^	16.1^b^	23.8^a^	15.0^c^	12.2^b^	22.8^a^	7.15^c^
Brevianamid	9.23^a^	5.58^b^	4.46^c^	23.2^a^	17.4^b^	6.99^c^	9.05^a^	7.46^b^	3.40^c^
Citreorosein	2.33^b^	<LOD	3.54^a^	4.84^b^	<LOD	7.39^a^	9.76^b^	13.3^a^	6.60^c^
Culmorin	18.6^b^	36.2^a^	0.15^c^	<LOD	23.5^a^	4.05^b^	15.8^b^	16.7^a^	5.04^c^
Cyclo(L-Pro-L-Tyr)	23.0^a^	8.08^b^	3.79^c^	26.6^a^	17.6^b^	5.64^c^	24.1^a^	14.7^b^	4.36^c^
Cyclo(L-Pro-L-Val)	19.1^a^	9.32^b^	3.03^c^	30.1^a^	21.0^b^	4.15^c^	19.8^a^	15.2^b^	1.79^c^
Deoxynivalenol	75.5^b^	75.8^a^	68.5^c^	67.4^c^	107.2^a^	75.1^b^	51.0^a^	40.9^c^	60.2^b^
DON-3-glucoside	2.82^b^	10.6^a^	<LOD	1.64^b^	3.35a	<LOD	1.86^b^	3.59^a^	<LOD
Emodin	1.94^a^	0.08	1.51^b^	5.33^a^	0.19	5.14^b^	9.06^a^	4.23^c^	5.86^b^
Enniatin A	<LOD	<LOD	<LOD	0.03^a^	<LOD	<LOD	<LOD	<LOD	<LOD
Enniatin A1	0.22^a^	<LOD	<LOD	0.16^a^	<LOD	<LOD	<LOD	<LOD	<LOD
Enniatin B	1.48^a^	0.02^c^	0.04^b^	0.77^a^	<LOD	0.08^b^	0.08^a^	<LOD	<LOD
Enniatin B1	0.87^a^	0.11^b^	<LOD	0.39^a^	<LOD	<LOD	0.06^a^	<LOD	<LOD
Enniatin B2	<LOD	<LOD	<LOD	<LOD	<LOD	<LOD	<LOD	<LOD	<LOD
Fumonisin B1	4.95^c^	5.45^a^	5.22^b^	8.15^a^	5.92^b^	5.56^c^	6.64^b^	7.34^a^	5.43^c^
HT-2 toxin	<LOD	2.61^a^	<LOD	<LOD	<LOD	<LOD	<LOD	<LOD	4.57^a^
Lotaustralin	10.4^a^	8.65^c^	8.95^b^	10.4^b^	11.2^a^	9.60^c^	9.67^a^	8.58^c^	9.08^b^
Moniliformin	1.25^b^	1.79^a^	<LOD	<LOQ	2.69^a^	<LOD	0.17	3.14^a^	1.28^b^
Monocerin	3.77^a^	<LOD	<LOD	1.33^a^	0.20^b^	<LOD	<LOD	<LOD	<LOD
Rugulusovin	2.36^a^	0.70	0.91^b^	2.63^a^	1.94^b^	1.50^c^	1.48^a^	0.60^c^	0.77^b^
T-2 toxin	1.26^b^	1.65^a^	<LOD	<LOD	0.44^b^	0.50^a^	1.79^b^	0.05^c^	13.1^a^
Tentoxin	0.31^b^	<LOD	0.33^a^	<LOD	<LOD	0.21a	<LOD	0.42^a^	<LOD
Tryptophol	61.6^a^	37.1^c^	38.3^b^	64.3^a^	41.6^b^	39.0^c^	53.6^a^	40.7^c^	41.2^b^

Values are means of triplicate. Values in the same row (2016–2018 for each variety) with different letters a–c are significantly different (*p* < 0.05). LOD and LOQ values are shown in Table S1, Supplementary Materials.

## Supplementary Materials

The following are available online at https://www.mdpi.com/2076-2607/7/11/532/s1, Table S1. LOD and LOQ values for detected metabolites.
