# Supplementary material for: Screening of Various Metabolites in Six Barley Varieties Grown under Natural Climatic Conditions (2016–2018)
Source: Microorganisms. 2019 Nov 6;7(11):532. doi: 10.3390/microorganisms7110532 (PMC6921044; doi:10.3390/microorganisms7110532)
Supplement: Supplementary file 1 [file microorganisms-07-00532-s001.pdf]

Supplementary Table S1 LOD and LOQ values for detected metabolites.

Toxin (μgkg^{-1})	LOD (μgkg^{-1})	LOQ (μgkg^{-1})
**15-Acetyldeoxynivalenol**	**0.5**	1.65
15-Hydroxyculmorin	0.05	0.165
15-Hydroxyculmoron	3	9.9
3-Acetyldeoxynivalenol	15	49.5
5-Hydroxyculmorin	5	16.5
Alternariol	0.1	0.33
Alternariolmethylether	0.04	0.132
Alternariolmethylether-glucoside	1	3.3
Altersetin	0.1	0.33
Anisomycin	0.01	0.033
Beauvericin	0.001	0.0033
Brevianamid	0.2	0.66
Chanoclavin	0.01	0.033
Chrysogin	0.05	0.165
Citreorosein	0.5	1.65
Culmorin	0.3	0.99
cyclo(L-Pro-L-Tyr)	0.1	0.33
cyclo(L-Pro-L-Val)	0.08	0.264
Deoxynivalenol	1.5	4.95
Dihydrogriseofulvin	0.15	0.495
DON-3-glucoside	0.4	1.32
Emodin	0.05	0.165
Enniatin A	0.002	0.0066
Enniatin A1	0.004	0.0132
Enniatin B	0.003	0.0099
Enniatin B1	0.005	0.0165
Enniatin B2	0.005	0.0165
Enniatin B3	0.0004	0.00132
Ergometrine	0.02	0.066
Fumonisin B1	0.40	1.32
Griseofulvin	0.15	0.495
HT-2 toxin	0.4	1.32
Kojic acid	20	66
Lotaustralin	0.4	1.32
Moniliformin	0.1	0.33
Monocerin	0.02	0.066
Rugulusovin	0.1	0.33
Sambucinol	0.2	0.66
T-2 toxin	0.1	0.33
Tentoxin	0.01	0.033
Tryptophol	5	16.5
